# The Intrinsic Nutrient Sensing Adipokinetic Hormone Producing Cells Function in Modulation of Metabolism, Activity, and Stress

**DOI:** 10.3390/ijms22147515

**Published:** 2021-07-13

**Authors:** Jonathan M. Nelson, Cecil J. Saunders, Erik C. Johnson

**Affiliations:** 1 Department of Biology, Wake Forest University, Winston-Salem, NC 27109, USA; nelsjm17@wfu.edu (J.M.N.); saundecj@wfu.edu (C.J.S.); 2 Center of Molecular Signaling, Wake Forest University, Winston-Salem, NC 27109, USA

**Keywords:** adipokinetic hormone, metabolism, nutrient sensing, AMP-activated protein kinase, ATP-gated potassium channels

## Abstract

All organisms confront the challenges of maintaining metabolic homeostasis in light of both variabilities in nutrient supplies and energetic costs of different physiologies and behaviors. While all cells are nutrient sensitive, only relative few cells within Metazoans are nutrient sensing cells. Nutrient sensing cells organize systemic behavioral and physiological responses to changing metabolic states. One group of cells present in the arthropods, is the adipokinetic hormone producing cells (APCs). APCs possess intrinsic nutrient sensors and receive contextual information regarding metabolic state through other endocrine connections. APCs express receptors for different hormones which modulate APC physiology and the secretion of the adipokinetic hormone (AKH). APCs are functionally similar to alpha cells in the mammalian pancreas and display a similar physiological organization. AKH release results in both hypertrehalosemia and hyperlipidemia through high affinity binding to the AKH receptor (AKHR). Another hallmark of AKH signaling is heightened locomotor activity, which accompanies starvation and is thought to enhance foraging. In this review, we discuss mechanisms of nutrient sensing and modulation of AKH release. Additionally, we compare the organization of AKH/AKHR signaling in different taxa. Lastly, we consider the signals that APCs integrate as well as recent experimental results that have expanded the functional repertoire of AKH signaling, further establishing this as both a metabolic and stress hormone.

## 1. Introduction

Changes in nutrient availability are thought to be a major selective force driving unique life history strategies such as migrations and hibernation states [1,2]. Additionally, different physiologies and behaviors require different energetic investments, for example, reproduction is energetically expensive and thought to represent a life history trade-off [3]. The mechanisms by which multicellular organisms balance dynamic changes in food and dynamic changes in energy investments require coordination of multiple systems. Subsequently, the ability to sense nutrient levels and organize a systemic response is a requirement of maintaining metabolic homeostasis. These nutrient sensing systems are typically found in the CNS and other endocrine associated tissues, as specific hormones are responsible for coordination of an organismal response in behaviors and physiologies. One such nutrient sensing center in Arthropods is the group of cells that express the adipokinetic hormone or APCs (AKH producing cells).

Adipokinetic hormone (AKH) was first isolated from the locust as a neurohormone that mobilized lipids during flight [4]. Subsequent to its initial discovery, AKH has been found widespread throughout the insects as well as in different Arthropod taxa. Specifically, a similar hormone was contemporaneously identified in decapod crustaceans that regulated pigment accumulation in the eyestalk. Red pigment concentrating hormone was found to be cross-reactive with insect AKHs, and later these molecules were found to possess similar primary sequences [5], despite having divergent functions. Even more interesting is that identification of the receptor, and subsequent studies probing AKH receptor evolution, implicates the AKH hormone signaling system as sharing ancestry with gonadotropin releasing hormone (GnRH) signaling. GnRH is widely found throughout the Metazoa, and primarily functions in reproductive maturation. Establishment of these evolutionary relationships are interesting because the actions of AKH have significant functional similarities to mammalian glucagon, and these similarities include regulatory mechanisms that control the nutrient-dependent secretion of these two hormones.

**Figure 1:**
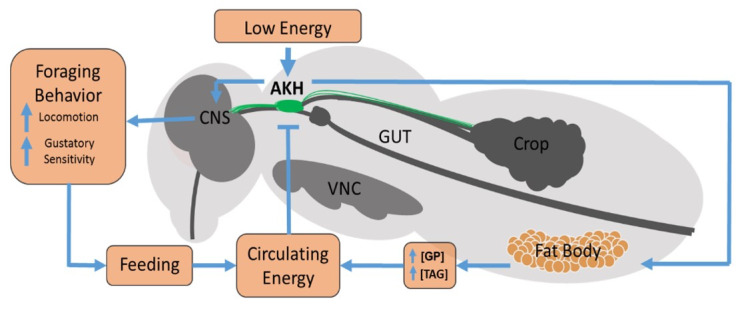
Model of AKH action. AKH producing cells (APCs) possess nutrient sensors which become active during low energy (high metabolic demand and/or low nutrient availability). This facilitates the release of AKH where it interacts with receptors located on the fat body and stimulates glycogen phosphorylase (GP) and TAG lipase to raise circulating levels of carbohydrate and lipid energy reserves. These inhibit the further release of AKH from APCs. AKH also binds to receptors present in the CNS, where AKH regulates other behavioral changes such as locomotor activity and is thought to act to modulate gustatory sensory information.

AKH canonically functions through selective binding to a specific receptor which ultimately leads to elevated activity of the glycogen phosphorylase enzyme and triglyceride lipases [6,7], which facilitates the mobilization of carbohydrate and lipid stores from the fat body. The fat body is the principal insect organ that stores both fat and sugar stores. Genetic experiments targeting AKH function in *Drosophila* show that AKH producing cells (APCs) are critical for normal behavioral and physiological responses to starvation conditions. Specifically, introduction of apoptotic induction genes to selectively kill APCs in *Drosophila* leads to an ensemble of different phenotypes, including increased longevity during starvation conditions compared to wild-type flies [8,9]. Additionally, these flies are typically larger, as they possess higher trehalose and triglyceride levels. Another interesting phenotype caused by this manipulation, and which is thought to explain the increased longevity during starvation, is the loss of starvation-induced hyperactivity [8,9]. Typically, starvation leads to elevated activity levels (in many different animals), which is thought to facilitate greater foraging efforts [10], and this response is absent in flies lacking APCs. Notably, other genetic manipulations indicate that the likely causative mechanism is the specific lack of the AKH gene. Specifically, the loss of the AKH and the loss of the receptor all produce the same phenotypes. Further confirmation comes from the observations, that ectopic expression of the AKH gene in the fat body provides genetic rescue to the APC ablations [9]. Collectively, these results suggest a model in which AKH is released during periods of low energy (high metabolic demand and/or low nutrient availability) where it acts upon the fat body to replenish circulating stores as well as in the CNS to facilitate concomitant behavioral changes (locomotor activity) (Figure 1).

In this review, we consider the organization of AKH producing cells (APCs), which possess nutrient sensors that become active during low energy (high metabolic demand and/or low nutrient availability). This change in metabolic demand facilitates the release of AKH, which interacts with receptors located on the fat body and stimulates glycogen phosphorylase and TAG lipase to raise circulating levels of reserves. These inhibit the further release of AKH from APCs. AKH also binds to receptors present in the CNS, where AKH regulates other behavioral changes such as locomotor activity and is thought to act to modulate gustatory sensory information.

We will next discuss the molecular organization of the AKH hormone in different taxa and discuss those relationships with specific functional attributes. We will then enumerate different intrinsic nutrient sensing mechanisms of APCs and speculate on other potential mechanisms. Next, we will discuss the signaling downstream of the AKH receptor and discuss its anatomic distribution and functional partitioning. Lastly, we will discuss endocrine modulators of APC activity, and speculate on whether AKH should be considered either as a metabolic or stress hormone. This is not meant to be an exhaustive review on the entire literature of AKH, but rather a current review of AKH functions that have recently been elucidated by genetic and molecular approaches, and so is heavily focused on *Drosophila*. 

## 2. Molecular Organization of the AKH Peptide

Adipokinetic Hormone is a small peptide hormone, and its primary sequence ranges from eight to 11 amino acids depending on organism and isoform. Uniformly, AKH peptides possesses an N-terminal pyro-glutamate and amidated C-terminus [11]. These modifications are, in general, thought to increase AKH peptide lifespan as it circulates through the hemolymph. The AKH mature hormone is processed by specific carboxypeptidases that recognize canonical dibasic cleavage sites. In *Drosophila*, mutations that impact the *amontodillo* gene, which encodes a prohormone convertase, results in animals with low circulating trehalose, which can be rescued by AKH under a heat shock promoter [12]. The exact mechanism for this AKH-deficient phenotype is unclear, as these phenotypes could arise from heightened AKH degradation in the hemolymph, or conversely from improper secretion due to improper peptide processing or, alternatively, reflect differences in hormone interactions with the receptor. However, these observations are consistent with other studies that show the C-terminal amidation of AKH is a crucial component of the peptide that is required for maximal receptor stimulation [13]. Furthermore, AKH appears to be degraded by specific peptidases associated with specific tissue types, such as the fat body, as opposed to circulating peptidases in the hemolymph [14], suggesting that AKH peptide degradation is occurring at the specific targets of AKH action. 

In insects, the mature AKH peptide hormone is produced in the neuroendocrine cells of the corpus cardiacum (CC), which is a glandular organ. The number of AKH isoforms varies with taxa, with some producing a single isoform [15] and others producing three or more [5]. The production of the various AKH mRNA transcripts can either arise from multiple genes presumably through a gene duplication, as is the case in the *Glossina morsitans* [16], or through alternative splicing, as has been predicted of the AKH gene in *Drosophila*. The functional difference between many of these variants currently remains under investigated, with much of the focus on metabolic pathways. In general, it appears that the AKH isoforms function in a redundant manner, possessing little, if any, functional distinctions from other endogenous forms of AKH (Table 1). However, we note that this generalization may not hold true as more AKH variants are thoroughly evaluated in different taxa.

However, in *Locusta migratoria* there are four AKH variants and these show different expression profiles, suggesting some novel differences among isoforms [4,17,21,22]. Specifically, AKH I and II levels increase in a linear manner during larval development, whereas in adults they maintain a stable ratio in respect to one another. The fourth isoform exists at very low levels [17]. Meanwhile, the expression of AKH III is present in greater abundance than that of the other isoforms during a small developmental window immediately prior to eclosion, after which it returns to its previous low level [23]. This expression profile may indicate a novel developmental function of AKH III in *Locusta*. Another example of expression differences in AKH isoforms is illustrated in the Bamboo Woolly Aphid, *Pseudoregma bambucicola*, in which differences in the ratio between ligand and receptor have been observed based on caste and the host plant [24].

In the mosquito *Aedes aegypti*, two separate and distinct genes encode AKH peptides and their expression demonstrate sexually dimorphic and temporal differences [25]. These instances suggest that AKH variants may play specialized roles. Perhaps the functional distinctions between the sexes and within developmental stages lie within differences in metabolic demand. It has been shown that, in *Manduca sexta,* the developmental transition from larvae to adults is accompanied with altered preference for nutrient substrates, specifically carbohydrates to lipids [26]. Furthermore, given the relatively higher metabolic costs of egg production compared to sperm, it seems probable that females require different metabolic needs. 

## 3. Nutrient Sensing by APCs

An interesting and fairly unique aspect of APC cell physiology is that APCs possess intrinsic nutrient sensors. While every cell is responsive to changing nutrient and energetic levels, APCs become activated by low intracellular energy as witnessed by an increase in free calcium levels in the cell [27,28]. This feature of APCs makes them of inherent interest to multiple fields as a cellular model for understanding coupling intracellular stimuli into organismal level responses in behavior and physiology. 

In APCs, there are two major mechanisms that are thought to function as nutrient “sensors”, which gives information about the metabolic state of the animal and translates that into release of the hormone. The first of which are ATP-gated potassium channels (K^+^ _ATP_ channels), which directly tie ATP availability to membrane potential and excitation-secretion [28,29]. The second known mechanism is the AMP-activated protein kinase, an enzyme that is activated by low energy content and alters APC cell physiology, enhancing AKH secretion [27]. We will discuss the evidence and mechanisms of each of these nutrient sensors and speculate on other potential metabolic sensors that may be present in APCs.

### 3.1. K^+^ _ATP_ Channels

K^+^ _ATP_ channels are multimeric ion channels composed of IRK (inwardly rectifying potassium) and SUR (sulfonylurea) subunits [30]. These channels are distributed in a wide array of different tissues, including mammalian pancreatic alpha and beta cells that release glucagon and insulin, respectively [31,32], as well as also being highly expressed in mammalian cardiac muscle [33]. These channels are also present in the mitochondria [34], where they are thought to gate potassium entry into the mitochondria as an important regulator of cellular respiration [35]. Indeed, much of what is known about the biochemistry and biophysics of these channels have emanated from using mammalian models. In mammals, there are multiple genes encoding different Sur and IRK channel subtypes, whereas in *Drosophila* there is a single gene encoding a SUR subunit (*Sur*), and three distinct genes encoding IRK channel subtypes [36]. In *Drosophila*, a single cell transcriptome from APCs verified the expression of all of these channel subtypes [29]. 

A genetic screen confirmed prior pharmacological studies implicating *Sur* as a critical regulator of APC cell physiology. Specifically, RNAi knockdown of the single *Sur* subunit in adult *Drosophila* phenocopied AKH loss of function variants, showing increased starvation lifespan, and reduced locomotor activity during starvation conditions [29]. Notably, APC cell viability and the capacity to synthesize the prohormone was unimpacted in animals expressing this genetic variant, leading to the conclusion that the likely explanation for the ensemble of phenotypes was a significant reduction in AKH secretion. In larval APCs, previous studies showed that tolbutamide, a SUR agonist, lead to elevated hemolymph levels of trehalose (consistent with elevated secreted rates of AKH), and such pharmacological action was dependent upon the presence of AKH cells [28]. 

K^+^ _ATP_ channels in pancreatic beta cells are connected to insulin release in mammals [37]. Specifically, when these cells have an abundance of ATP, the excess ATP closes the K^+^ _ATP_ channels, which are constitutively open. This closing leads to cellular depolarization as the potassium leak current is stopped. This depolarization leads to the opening of voltage-gated calcium channels and the subsequent release of insulin, which will have the net effect of lowering blood sugar in mammals [38]. However, this straightforward connection between K^+^ _ATP_ channel activation and hormone release (for both glucagon and AKH) cannot be the case. Release of these hormones are in response to hypoglycemic conditions, and the β cell model of excitation-secretion coupling would have the opposite effect on cell excitability for both APCs and mammalian pancreatic alpha cells. For mammals, various solutions for this “alpha cell conundrum” have been offered and include paracrine regulation of glucagon release, different equilibrium potentials for α cells, a lack of K^+^_ATP_ channel gating in hormone release but not calcium entry, and differential modulation of spontaneous activity [39]. While this aspect of APC physiology has not been completely explored, the rationale to do so may be to offer insight into a complicated mechanism of how K^+^ _ATP_ channels fit into the picture of regulated the secretion of a hyperglycemic hormone, such as glucagon and AKH. 

**Figure 2:**
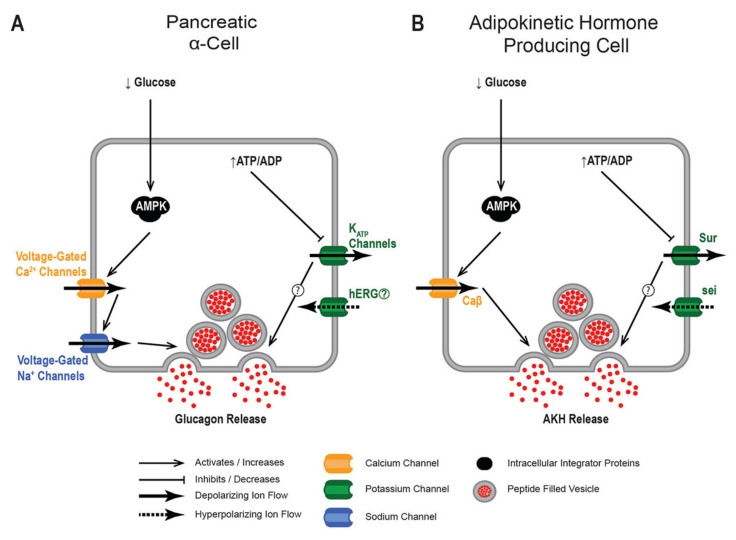
Functional similarities between physiological mechanisms of (**A**) pancreatic α cells and (**B**) APCs. APCs and pancreatic α cells possess many similar mechanisms that control the secretion of AKH and glucagon, respectively. Notably, AMPK and K^+^_ATP_ channels are present in both cell lineages and function as nutrient sensors. The exact mechanism for how K^+^_ATP_ channels function as a nutrient sensor is less clear in both cell types. Furthermore, the identities of many of the ion channels that regulate the excitability of each tissue are similar (specifically calcium and potassium channels).

While the AKH hormone shares evolutionary relationship with the GnRH family members [40], what is interesting is the common elements that are shared in APCs and pancreatic alpha cell physiologies as these suggest a convergent evolution regarding nutrient sensing mechanisms. We speculate that there may be a limited repertoire of nutrient sensing molecules that have been co-opted in specific cell lineages. Notably, APCs and pancreatic alpha cells are derived from different embryological anlagen [41], implicating that APCs and pancreatic alpha cells are not derived from a common ancestor. The sc-transcriptome of APCs implicate other channel types, including voltage-gated calcium channels and potassium channels, as being critical in setting the dynamics of APC excitability [29]. Comparisons of cellular physiology between this cell type and the pancreatic alpha cells are remarkably similar and suggest a convergent evolution (Figure 2), furthering the argument that increased study of APCs could inform aspects of pancreatic alpha cell biology and human disease, as well as offer insight into the physiology of other nutrient sensing tissues. 

### 3.2. The AMP-Activated Protein Kinase (AMPK)

The other nutrient sensor present in APCs, is the AMP-activated protein kinase (AMPK). AMPK is widely distributed throughout the metazoan and is widely recognized as a cellular mechanism mediating energetic homeostasis, by virtue of its activation by the end product of ATP hydrolysis [42,43]. AMPK is connected to many disparate intracellular signaling pathways, which makes sense given the broad extent of how low energy impacts different intracellular energy utilization programs. The functional enzyme exists as a heterotrimer possessing a catalytic alpha (α), a regulatory gamma (γ), and a scaffolding beta subunit (β) [44,45]. In mammals, multiple genes encode each of the subunits, which form different heterotrimeric complexes [46,47,48]. Thus, despite intense scrutiny of the roles of AMPK in mammals, the complete organismal context of AMPK function remains poorly understood. In *Drosophila*, a single gene encodes each subunit, and clear homologs of each subunit are present and display 60%, 62%, and 62% identity with human α, β, and γ subunits, respectively. *Drosophila* AMPK is highly similar to mammalian AMPK, as it is formed via a heterotrimeric complex, is activated by AMP, and has many of the same targets, including acetyl-CoA carboxylase (ACC) [49]. Consequently, a genetic approach to dissect AMPK function is both feasible and practical in *Drosophila*. 

Activation of AMPK by AMP leads to the phosphorylation of many targets, including acetyl CoA carboxylase (ACC) and the peroxisome proliferator activated receptor (PPAR), which has the net effect of diverting energy away from energy requiring processes and towards a conservation or energy production mode [50]. Indeed, the downstream signaling pathways with which AMPK intersects is substantial. Genetic modulation of AMPK activity in various model systems suggests that AMPK may represent an underlying mechanism to increase longevity as a consequence of dietary restriction [51], and also that AMPK activity may be responsible for the benefits of exercise [52]. Additionally, pharmacological agents targeting AMPK activity have been developed as therapeutics for treatment of human pathologies associated with diabetes [53]. 

In *Drosophila*, ubiquitous knockdown of AMPK expression leads to a persistent starvation phenotype, as measured by a number of different physiological and behavioral measures, in both larvae and adult stages [54,55]. Additionally, other systemic behavioral consequences are hyperphagia, reduced locomotor activity, and a reduction of stored triglycerides [54]. These results suggest, akin to the mammalian literature, cell context specifically dictates AMPK function. For example, in mammals, AMPK signaling leads to reduced insulin release [56,57], while simultaneously activating glucagon release [58]. Likewise, in hypothalamic neurons, AMPK stimulation leads to neuropeptide Y release that leads to feeding behaviors [59]. In *Drosophila*, reduction of AMPK function either by introduction of a dominant negative construct or RNAi elements, lead to a partial phenocopy of AKH loss of function variants [27]. The behavioral phenotypes are supported by examination of AMPK action on APC excitability, specifically that reduced AMPK function blunts but does not eliminate the intrinsic activation of APCs to low trehalose levels. Interestingly, activation of AMPK under replete conditions using the AMPK agonist, AICAR, increases calcium levels independent of any energetic changes, suggesting that altered APC excitability is the mechanism for AMPK action [27]. It is unclear whether AMPK phosphorylates the complement of ion channels present in APCs [29], and of specific interest is whether AMPK interacts with K^+^ _ATP_ channels or whether these two different nutrient sensors are independent. The partial phenotypes associated with AMPK loss of function strongly suggest additional nutrient sensing mechanisms.

In *Drosophila*, larval AKH cells appear to require extracellular trehalose for secretion. Specifically, explanted larval APCs had higher levels of AKH immunostaining when placed in media lacking trehalose as compared to similar groups of cells placed in media containing trehalose. The authors concluded this was caused by reduced secretion as the cells were co-incubated with a protein synthesis inhibitor to rule out altered production. While this study concluded that AKH cells were the source of sugar detection that led to the subsequent secretion of ILPs (insulin-like peptides) in *Drosophila* [60], the notion of AKH release as a function of trehalose is somewhat counterintuitive. It is unclear if this is an aspect of a complex dose relationship of trehalose acting on these cells or a developmental aspect. It is tempting to suggest that the parallel findings in APCs and pancreatic alpha cells, showing glucose-dependent glucagon secretion [61] might be an underlying mechanism that explains the alpha cell conundrum. Likewise, this same study suggested that nutrient sensing mechanisms in the fat body relay information back to APCs, suggesting a complex interplay between APCs and other potential nutrient sensing cell populations [60]. 

## 4. AKH Receptor Distribution and Signaling

**Figure 3:**
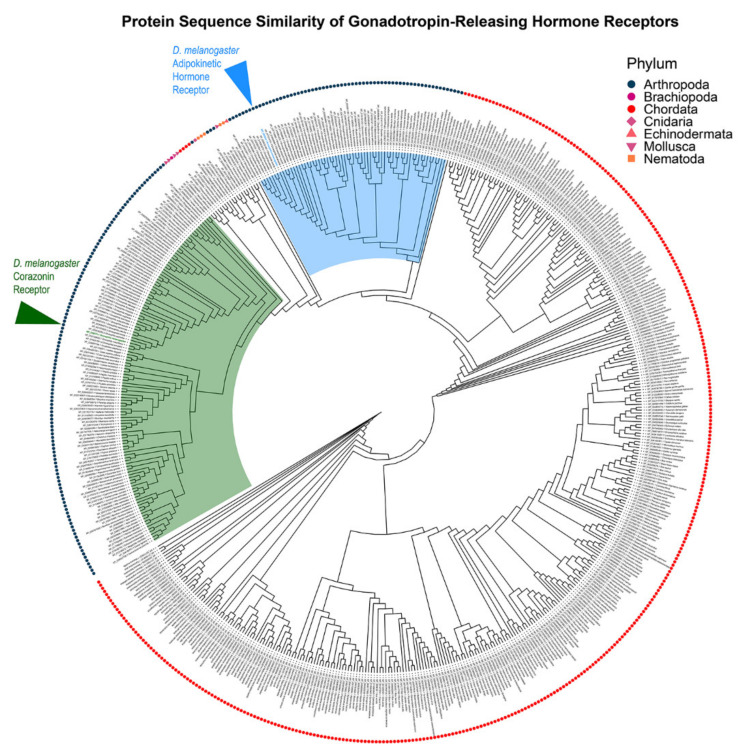
AKH signaling shares homology with gonadotropin releasing hormone signaling (GnRH). Cladogram depicting the protein sequence similarity between *D. melanogaster’s* AKH receptor (blue arrowhead), corazonin receptor (green arrowhead), and GnRH receptor reference sequences from NCBI protein. Arthropod GnRH receptors formed two major clusters, one containing AKH-like GnRH receptors (blue wedge) and the other containing corazonin-like GnRH receptors (green wedge). For each receptor, the source phylum is indicated by a symbol and labeled with the sequence accession number and species name. The source code and files for construction of the tree are available in a GitHub repository [74].

The AKH receptor is a member of the class A rhodopsin G protein-coupled receptor family [62], and was first identified through a heterologous expression system expressing *Drosophila* cDNAs for receptor molecules [63,64]. Expression of cDNA corresponding to the *CG11325* gene annotation confers AKH-evoked responses in calcium in vertebrate cell lines [63,64]. In addition to this functional characterization, genetic manipulations of *CG11325* have consistently produced the same set of behavioral and physiological phenotypes as either genetic loss of the AKH hormone or genetic ablation of the APCs [65,66]. This further confirms the identity of this gene encoding an AKH-receptor (AKHR). Subsequent to the discovery of AKHR, homologs have been found in multiple insect and crustacean taxa [25,67,68]. Interestingly, a phylogenetic analysis of this *Drosophila* gene and other insect homologs firmly establish an evolutionary relationship with the GnRH receptor subfamily [62] (Figure 3). In these heterologous expression systems, AKHR couples to both calcium and cAMP second messenger systems [68,69], which is consistent with observation of AKH evoked changes in different insects [18,70,71]. Specifically, that exogenous application of AKH results in the activation of PLC, resulting in an increase of internal calcium. However, AKH binding has been shown to activate adenylyl cyclase and subsequent increases in cAMP [72]. These molecular details of AKH signaling are reviewed in greater detail by Gäde and Auerswald [73].

In the mosquito, *Aedes aegypti*, there are two splice variants of AKHR [25] that differ in ligand selectivity [75]. It is plausible that such specificity arose to allow different signaling cascades among different cell types possessing the receptors. However, not all organisms have adapted this approach and the number of AKH receptors varies between species. In stark contrast to this, in *S. gregaria* AKHR binds just as readily to AKH variants of other species as it does to endogenous AKH isoforms [75]. In *L. migratoria* and *S. gregaria* there is a single AKH receptor. Interestingly, in both species of locust this single receptor binds to multiple endogenous isoforms of AKH. Within *Drosophila*, there is a single gene encoding AKHR and sequence analysis predicts the presence of four splice variants. Three of these give rise to the same amino acid sequence and vary in the UTR regions. The fourth, AKHR-RC, possesses 12 unique amino acids at its C-terminus. It is unclear what impact, if any, this may have on the receptor. To date, there have been no studies examining the presence or function of AKHR-RC in *Drosophila*.

AKH receptor variants may serve to promote alternative signaling pathways. Receptor isoforms of the tsetse fly differ in the sequence of the C-terminal tail. Similarly, GnRH receptor isoforms commonly show differences in the length of the C-terminus, which impacts multiple aspects of receptor physiology [76,77,78], and interestingly is a common variant among different GnRH receptor isoforms [79]. Quantitative differences in the amount of intracellular calcium release through signaling of the receptor isoforms differs by 2.5-fold when activated by the AKH isoforms, with roughly a 1.5-fold difference between the two neuropeptides [24]. *Aedes aegypti* possesses two AKHR splice variants. Again, these variants differ in their C-terminal end, mainly in the form of truncation, and the major difference appears to be expression patterns [25]. However, within *A. aegypti*, receptor isoforms seem to be co-expressed in many of the same regions, other than the ovaries, where only one receptor isoform is present [25]. The presence of a single variant of AKHR within the ovaries is particularly interesting, as it may suggest a novel communication between the metabolic state of the organism and reproductive function that is mediated via AKH. 

Examination of AKH receptor distribution has the ability to pinpoint anatomical substrates of AKH function, as well as suggest novel functions. Within *Drosophila* and other insects, prominent clusters of AKHR+ cells are located within the fat bodies of larvae and adults [80]. It is these cells that play a pivotal role in energy mobilization through modulation of glycogen phosphorylase and TAG lipase activity [7,81]. In addition to the expected expression in the fat body, AKHR is expressed in octopaminergic neurons of the subesophageal zone (SEZ) [82], which is a critical central component that influences locomotor behaviors in an AKH-dependent manner. In the same region of the brain, four neurons possessing AKHR play a key role in regulation of feeding and drinking [19]. Other studies have provided further evidence regarding the presence of AKH receptive neurons within the SEZ, specifically within the subesophageal ganglion, and have identified many as attractive-gustatory neurons [65]. AKH recipient neurons have also been shown to innervate the antennal lobe where they alter preferences to pheromones [83]. The loss of the same neurons causes significant changes in female production of the pheromones 7-tricosene and 7,11-heptacosadiene based on starvation status [83]. Additionally, changes in AKHR expression as a consequence of hunger have been identified in the antennae as well [84]. More recently, AKHR-IA, but not AKHR-II is expressed within the ovaries of *Aedes aegypti* [85], perhaps indicating a novel role of AKH in reproduction. An interesting study reported that APCs are UV light sensitive, through specific expression of a dTRPA1 isoform, suggesting a role in APCs being critical in stress responses [86].

### Hormonal Inputs into APCs

While the demonstration that explanted larval and adult APCs increase intracellular calcium levels during a transition between high and low sugar levels implicates intrinsic nutrient sensing mechanisms, it is reasonable to assume that, in vivo, APCs also receive inputs from other nutrient sensing centers. Likewise, APCs presumably are modulated by other hormones that give contextual information but not nutrient sensing per se. The challenge is to identify which hormones alter APC cell physiology, and then to determine the nature of this modulatory signal.

Many different hormones and peptides have been identified in various bioassays in locusts. Specifically, application of tachykinin peptides, crustacean cardioactive peptide (CCAP), and members of the myosuppressin peptides have all been shown to modulate AKH titers in the locust [87,88,89]. Furthermore, other transmitters such as octopamine were also found to modulate APC physiology and the secretion of the AKH hormone, and, in this case, is thought to connect AKH to the locomotor effects, as octopamine has been previously demonstrated to modulate locomotor behaviors in many different insect species [90,91,92,93]. Interestingly, the interplay between octopamine and AKH on locomotor profiles are evident under replete conditions, implicating a modulatory role in metabolism.

In *Drosophila*, the allatostatin-A (Ast-A) peptides are known to regulate feeding behaviors and are ancestrally related to mammalian galanin peptides. Ast-A modulates APC physiology, and APC specific introduction of an RNAi element targeting the AstA R-2 leads to altered starvation sensitivity and triglyceride and trehalose levels. This is consistent with a model in which AstA facilitates AKH release [94]. Notably, AstA also influences insulin producing cells (IPCs) in a similar fashion [94]. It is currently unknown whether the neurons or endocrine cells that express this peptide hormone also possess intrinsic nutrient sensors. However, given the functions of this peptide in other starvation-related behaviors (specifically, feeding), it is consistent that this peptide would also trigger AKH release and one may predict that Ast-A expressing cells express nutrient sensors. Furthermore, Ast-A peptides share homology with the mammalian galanin peptides, and one function of mammalian galanin signaling is enteric gut nutrient sensing [95,96].

A pair of glucose-sensitive neurons inhibit AKH release and these neurons express both the corazonin and short NPF neuropeptide hormones [97]. The apparent transmitter is the short NPF peptide, which also has been shown to regulate IPCs as well [98]. This suggests that a common regulatory theme in these metabolic networks is that hormones that regulate APCs are likely to regulate IPCs as well and vice versa. Along those lines, we recently reported that the circadian hormone, PDF, modulates APCs [99] and previous descriptions have found this peptide also targets IPCs [100]. However, the above finding suggests that endocrine and intracellular metabolic context presumably is an important aspect that governs AKH release. In addition, we have also found that APCs express multiple receptors for dopamine, and the signals elicited from these DA receptors appear to be context-dependent [93,99].

## 5. Concluding Remarks

**Figure 4:**
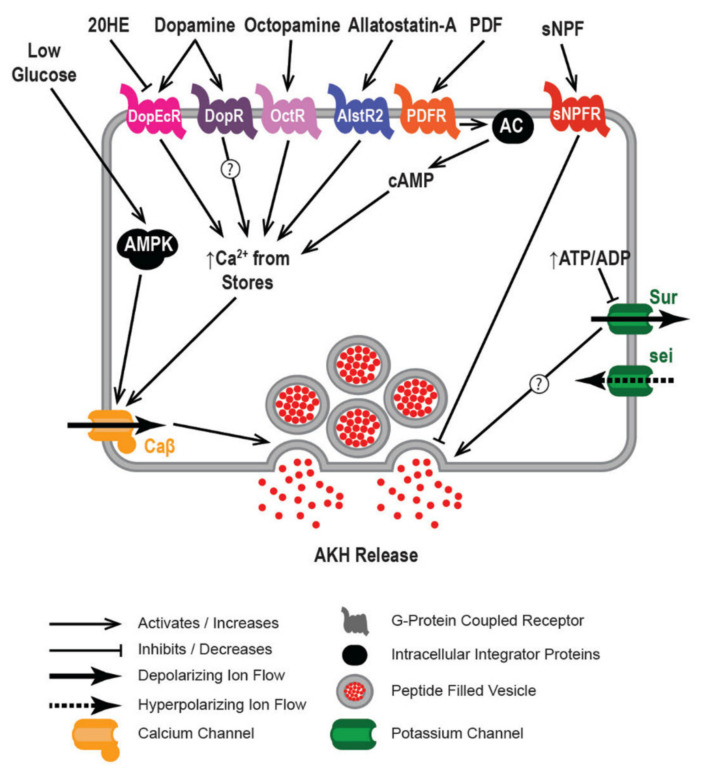
Model of APC physiology. (Top) AKH cells receive hormonal input via GPCRs. Dopamine leads to the activation of multiple dopamine receptors present in AKH cells. These receptors in turn directly lead to the influx of calcium in a sugar dependent manner. Ecdysone application blocks dopamine-dependent responses. PDF acts on AKH cells via PDFR and in turn leads to increased production of cAMP through an unidentified adenylate cyclase. Presumably increases in cAMP modulate calcium influx. (Middle) AMPK is activated by low sugar and in turn leads to calcium influx. (Bottom) AKH cells express multiple ion channels that regulate membrane excitability. Notably the Sur subunit of K^+^_ATP_ channels responds to changes in cellular ATP. Ca-Beta is a regulatory subunit of the functional calcium channel. *sei* modulates potassium influx and baseline membrane potential.

AKH has long been appreciated as a metabolic hormone, as an extensive literature has documented its direct impact on glycogen phosphorylase and TAG lipase in a wide variety of different Arthropod taxa. Consistent with such an idea is the observation that the APCs possess nutrient-sensing mechanisms and share some physiological features with mammalian pancreatic alpha cells. Furthermore, recent work has begun tackling neuroendocrine circuits that modulate AKH release and many of these other hormones are likewise involved in nutrient sensing, suggesting a complex network of signals (Figure 4). Of particular interest is that, while the glucagon and AKH are evolutionarily isolated, there appears to be convergence in the architecture of cell physiology between APCs and pancreatic alpha cells. The evolutionary relationship between GnRH and AKH is also interesting. One is tempted to speculate that ancestral GnRHs, which are primarily responsible for fueling reproduction and many GnRH expressing cells express nutrient sensors [101], became co-opted in the case of AKH to fulfill a more metabolic role. 

AKH may also be considered a “stress” hormone, although one wonders about the utility about such a designation. It is clear that AKH impacts physiologies and behaviors that are altered during many different stressful environments. The enhanced locomotion downstream of AKH release would facilitate the removal of the organism from unfavorable environments. Additionally, AKH has inherent anti-oxidative aspects [74,102,103,104] and, though we did not discuss this topic due to space constraints and having a different focus for the review, such observations do strengthen the conceptual argument that perhaps AKH is predominantly a stress hormone that has metabolic functions. We recognize that this argument may be entirely semantic; however, regardless may form a conceptual framework for unifying AKH functions. 

Mechanisms of APC nutrient sensing still garner significant interest and we suspect more information will be learned about the precise mechanisms of nutrient sensing interacting with cell excitability components. Furthermore, the neural and endocrine circuits that regulate AKH and those that AKH participates in are a continued area of study. Such information is likely to broaden our perspectives of the hormone and implicate novel functions in addition to garnering deeper insight into how this important signaling system operates. 

## Figures and Tables

**Table 1:** AKH isoforms possess conserved function. Examination of the physiological effect of each AKH isoform as reported in six difference species [9,17,18,19,20]. In general, it appears that many AKH isoforms are redundant and serve to initiate carbohydrate and lipid metabolism. Filled boxes indicate that the isoform has an effect on a given phenotype, empty boxes indicate a lack of data, and checked box indicates that evidence supports a lack of effect.

Species	Name	Sequence	Lipid Metabolism	Carbohydrate Metabolism	Locomotion/Flight	Cardiac Function
*Bombyx mori*	AKH I	pQLTFTSSWG-NH_2_				
*Bombyx mori*	AKH II	pQLTFTPGWG-NH_2_				
*Bombyx mori*	AKH III	pQITFSRDWSG-NH_2_				
*Drosophila melanogaster*	Drome-AKH	pQLTFSPDW-NH_2_				
*Periplaneta americana*	Peram-AKH-I	pQVNFSPNW-NH_2_				
*Periplaneta americana*	Peram-AKH-II	pQLTFTPNW-NH_2_			X	
*Locusta migratoria, Schistocera gregaria*	Locmi-AKH-I	pQLNFTPNWGT-NH_2_				
*Locusta migratoria*	Locmi-AKH-II	pQLNFSAGW-NH_2_				
*Locusta migratoria*	Locmi-AKH-III	pELTFTPSW-NH_2_				
*Locusta migratoria, Schistocera gregaria*	Locmi-AKH-IV	pQLNFSTGW-NH_2_				
*Schistocera gregaria*	Schgr-AKH-II	pQLNFSTGW-NH_2_				
*Manduca sexta*	Manse-AKH	pQLTFTSSWG-NH_2_				

## Data Availability

The source code and files for construction of the tree depicted in Figure 3 are available in a GitHub repository [20].
